# Role of the NLRP3 inflammasome in the transient release of IL-1β induced by monosodium urate crystals in human fibroblast-like synoviocytes

**DOI:** 10.1186/s12950-015-0070-7

**Published:** 2015-04-10

**Authors:** Shu-cong Zheng, Xiao-xia Zhu, Yu Xue, Li-hong Zhang, He-jian Zou, Jian-hua Qiu, Qiong Liu

**Affiliations:** Division of Rheumatology, Huashan Hospital, Fudan University, Shanghai, China; Institute of Rheumatology, Immunology and Allergy, Fudan University, Shanghai, China; Department of Anatomy, Histology and Embryology, Shanghai Medical College of Fudan University, 138 Yixueyuan Road, Shanghai, 200032 PR China; Division of Emergency Medicine, Boston Children’s Hospital, Harvard Medical School, Boston, MA USA; Key Laboratory of Medical Imaging Computing and Computer Assisted Intervention of Shanghai, 138 Yixueyuan Road, Shanghai, 200032 PR China

**Keywords:** MSU, NLRP3, Inflammasome, IL-1β, Synoviocytes

## Abstract

**Background:**

To investigate whether monosodium urate (MSU) crystals induce interleukin (IL)-1β in human fibroblast-like synoviocytes (FLS), and whether the NLRP3 inflammasome is involved in the inflammatory mechanism.

**Methods:**

Human FLS isolated from explants of synovial tissue were stimulated with MSU crystals (0.001 to 0.5 mg/ml) for different time course (6 hours to 48 hours). The expressions of IL-1β, IL-6, TNF-α and NLRP3 were evaluated with ELISA, Western blot and quantitative real-time PCR.

**Results:**

Exposure of FLS to MSU crystals transiently induced a significant increase in IL-1β expression in culture medium with a peak at 6 h. The mRNA level of IL-1β in the FLS cells had a similar pattern at this time point. Changes in IL-6 and TNF-α expression were not observed. Simultaneously, intercellular pro-IL-1β was detected at 6 h. Furthermore, MSU crystals also induced NLRP3 mRNA and protein expression at 6 h to 48 h after MSU treatment.

**Conclusions:**

MSU crystals directly increased IL-1β and intercellular NLRP3 expression in FLS cells. It is suggested that the NLRP3 inflammasome may be associated with IL-1β in FLS treated with MSU. Altogether, MSU could induce production and release of IL-1β through the NLRP3 inflammasome in human synoviocytes.

**Electronic supplementary material:**

The online version of this article (doi:10.1186/s12950-015-0070-7) contains supplementary material, which is available to authorized users.

## Background

Gout is one of inflammatory arthritis due to deposition of monosodium urate (MSU) crystals in synovial fluid and joints [1]. As demonstrated in vivo, MSU crystals cause inflammation. When MSU crystals were injected into the peritoneum in an animal model of acute gout, it induced the production of proinflammatory cytokines such as interleukin-1β (IL-1β) [2].

Although MSU is identified as a causative agent in gout [3,4], the mechanisms underlying MSU crystal-induced inflammation have only recently begun to be understood [3,5]. Pro-inflammatory cytokines play a critical role in the inflammatory reaction induced by MSU-crystals [6,7]. It is suggested by several studies that IL-1β, the pathological hallmark of the acute inflammatory attack, is a key regulatory pro-inflammatory cytokine in gout, which promotes a neutrophil influx into the synovium and joint fluid [8,9]. Moreover, IL-1β also plays a crucial role in driving the transition from the acute phase of arthritis to the chronic irreversible phase [10].

IL-1β-dependent inflammation, occurring in a number of diseases in addition to gout, depends on the MSU-induced formation of a macromolecular nucleotide-binding domain-like receptor protein 3 (NLRP3) inflammasome complex [3,11-13]. The NLRP3 (also known as NALP3) inflammasome has been shown to form through homotypic interactions between the CARD and PYD domains of NLRP3, Pyrin and apoptosis-associated speck like protein (ASC). After the NLRP3 inflammasome is formed, it converts procaspase-1 to active caspase-1, which in turn cleaves pro-IL-1β to active IL-1β. Although MSU has been described as a causative agent in gout for more than 100 years [14], its mechanism of action was not understood until the discovery of NLRP3 a decade ago [15].

Accumulating evidence suggests that MSU crystal-induced inflammation has undergone phagocytosis-activated the NLRP3 inflammasome, which leads to secretion of IL-1β from resident macrophages [16-18]. In turn, this secretion can increase the production of IL-1β and other inflammatory mediators further, and promote the activation of synovial lining cells and phagocytes [3,19]. Recent studies show that MSU crystals induce synoviocytes to release CCL2 (monocyte chemoattractant protein-1; MCP-1) and recruit monocytes/macrophages to joints [20]. However, it is unclear whether MSU causes synoviocytes to produce IL-1β, and if so, what the potential mechanisms are. The aim of the present study is to address whether synoviocytes can activate transcriptional levels of IL-1β, and secrete IL-1β after exposure to MSU crystals. An additional aim is to explore the potential underlying molecular mechanisms.

## Materials

### FLS isolation and culture

The study was approved by the Ethics Committees of Huashan Hospital, Fudan University. Synovial tissues were obtained from one patient, who had joint replacement due to idiopathic femoral head necrosis. Synovial fibroblasts were isolated from the synovial tissues by enzymatic digestion. FLS were isolated from tissue explants, as previously described [21]. In brief, synovium tissues were rinsed several times in PBS, minced into ~1-mm pieces, placed in T25 flasks (Falcon, USA), and maintained in DMEM supplemented with 10% heat-inactivated fetal calf serum (FCS), 50 μg/ml streptomycin, 50 U/ml penicillin, and 2 mmol/L glutamine (10% FCS medium). At confluence, cells were harvested (trypsin/EDTA) and seeded into new flasks. All experiments were carried out with passage 4 through 8 FLS.

### Reagents

MSU crystals were purchased from Alexis (Enzo Life Sciences, USA). Cell culture reagents including media, phosphate buffered saline (PBS), and Hank’s balanced salts solution (HBSS), penicillin-streptomycin, glutamax, and fetal calf serum (FCS) were obtained from Invitrogen (USA). Polyclonal anti-pro-IL-1β, anti-NLRP3 antibody was purchased from Santa Cruz Biotechnology (Santa Cruz, USA).

### Cytokine detection by ELISA

FLS were plated at 2.0 × 10^4^ cells/well in a 24-well plate. When they reached subconfluency, the medium was removed, and fresh medium was applied along with MSU (1, 10, 50, 100, 200, 500 ug/ml). The supernatants were collected after stimulation with MSU for 6 h, 12 h, 24 h and 48 h respectively. IL-1β, IL-6, and TNFα levels in the supernatants were measured by ELISA (R&D Systems, USA) according to the manufacturer’s protocol.

### Quantitative real-time PCR

Total RNA was extracted using RNA Lyzol reagent (EXcell Bio, Shanghai, China). cDNA was synthesized with the Rever TraAceHqPCR RT Kit (TOYOBO. CO, TLD, Japan). Quantitative real-time PCR was performed on a 7500 Fast Real-Time PCR System (AB Applied Biosystems, USA) using SYBRH Green Realtime PCR Master Mix (TOYOBO. CO, TLD, Japan). The specificity of amplification was assessed for each sample by melting curve analysis. Relative quantification was performed using standard curve analysis. The quantification data are presented as a ratio to the control level. The Homo sapiens (hs) gene specific primers used were as follows: IL-1β, 5′-TTGTTGCTCCATATCCTGTCC-3′ (forward) and 5′-CACATGGGATAACGAGGCTT-3′ (reverse); IL-6, 5′-GGAGACTTGCCTGGTGAA-3′ (forward) and 5′-GCATTTGTGGTTGGGTCA-3′ (reverse); TNFα, 5′-CACTAAGAATTCAAACTGGGGC-3′ (forward) and 5′- GAGGAAGGCCTAAGGTCCAC-3′ (reverse); NLRP3, 5′-TAAAGAGATGAGCCGAAGTGGG-3′ (forward) and 5′-TCAATGCTGTCTTCCTGGCA-3′ (reverse); GAPDH, 5′-ATGACCCCTTCATTGACC-3′ (forward) and antisense 5′-GAAGATGGTGATGGGATTTC-3′ (reverse).

### Western blot analysis

FLS were seeded in 6-well culture plates at a density of 1 × 10^6^ cells/well. The cells were allowed to adhere for 24 hours, and then the cells were cultured in the medium with 2% FCS and 50 μg/ml MSU crystals for 6 hours or 48 hours. The cells were disrupted in lysis buffer (20 mM Tris, pH 7.5, 150 mM NaCl, 1 mM EDTA, 1 mM EGTA, 1% Triton X-100, 2.5 mM sodium pyrophosphate, and 1 mM b-glycerophosphate) with 1 mM PMSF, 1 mg/ml leupeptin, and 1 mM sodium orthovanadate (Sigma, USA). The concentrations of the extracted proteins were measured using a BCA Protein Assay Kit (Thermo Fisher Scientific, USA). Samples (50 μg of total protein) were dissolved with equal volume of loading buffer (0.1 M Tris–HCl buffer (pH 6.8) containing 0.2 M DTT, 4% SDS, 20% glycerol and 0.1% bromophenol blue), separated on 10% SDS-PAGE and then electrotransferred at 100 V to Immun-Blot PVDF membrane for 1 hour at 4°C. Membranes were blocked in TBST containing 5% non-fat milk overnight at 4°C before incubation for 2 h at room temperature with primary antibodies diluted in TBST containing 5% BSA. Blots were washed extensively in TBST and incubated with secondary antibodies in TBST/1.25% BSA for 1 h at room temperature. The signal was detected by an enhanced chemiluminescence method (ECL kit, Amersham), and exposed to Kodak X-OMAT film (Eastman Kodak, Rochester, NY, U.S.A.). The intensity of the selected bands was captured and analyzed using GeneSnap Image Analysis Software (Syngene, U.K.). We used rabbit polyclonal anti-human NLRP3 antibody (Santa Cruz Biotechnology, Santa Cruz, CA, USA), anti-human pro-IL1-β (Santa Cruz Biotechnology, Santa Cruz, CA, USA) and rabbit polyclonal anti-human GAPDH antibody (Cell Signaling, Danvers, MA) as primary antibody and anti-rabbit IgG HRP-linked antibody (Cell Signaling, USA) as secondary antibody.

### Data analysis

Data are presented as mean ± S.E.M. and analyzed by SPSS 11.0. Repeated measures analysis of variance (ANOVA) followed by S-N-K test were used for post-hoc analysis of differences between groups. P < 0.05 was considered a statistically significant difference.

## Results

### Effects of MSU on the release of proinflammatory cytokines IL-1β, TNFa and IL-6 from FLS

**Figure 1:**
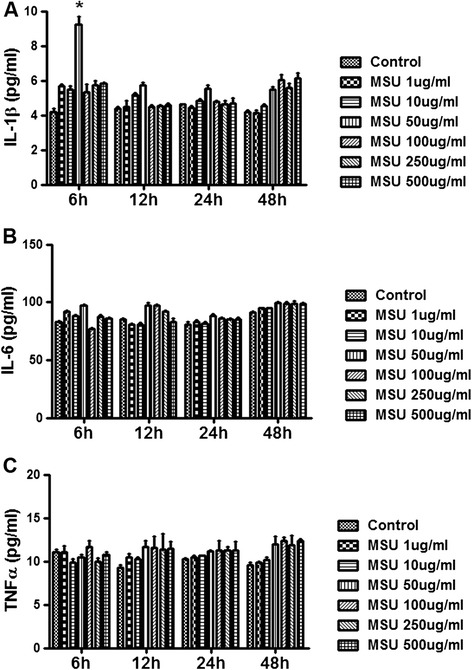
**MSU induced IL-1** **β production in FLS.** FLS were stimulated with 1 ug/ml, 10 ug/ml, 50 ug/ml, 100 ug/ml, 250 ug/ml and 500 ug/ml of MSU at 6 h, 12 h, 24 h and 48 h respectively. The control group is treated with PBS, which is used to dilute different dosage of MSU. Supernatants were collected for IL-1β, TNF-α and IL-6 protein detection by ELISA. The concentration of MSU 50 ug/ml induced the significant increase of IL-1β in the supernatants collected after 6 hours MSU exposure (**A**, p<0.05). There is no significant difference for the levels of IL-6 **(B)** and TNF-α **(C)** in the supernatant of FLS exposed to different concentration MSU at the four time courses. Data presented are mean ± S.E.M. *represents P < 0.05 in comparison with control during statistical analysis.

We first compared IL-1β, TNFα and IL-6 levels at different time points in the culture media after FLS cells were exposed to different concentrations of MSU crystals. The levels of IL-1β, TNF-α and IL-6 triggered by 1 ug/ml, 10 ug/ml, 50 ug/ml, 100 ug/ml, 250 ug/ml and 500 ug/ml of MSU at 6 h, 12 h, 24 h and 48 h respectively are shown in Figure 1. The results indicated that 50 ug/ml of MSU induced a significant increase in IL-1β after 6 hours, but not at other time points (Figure 1A). The result was re-confirmed by using serial MSU concentrations close to 50 ug/ml (Additional file 1: Figure S1). Furthermore, there were no significant differences in levels of IL-6 (Figure 1B) and TNFα (Figure 1C) in FLS exposed to different concentrations of MSU at all exposure periods.

### IL-1β, TNFa and IL-6 mRNA expression in MSU treated FLS

**Figure 2:**
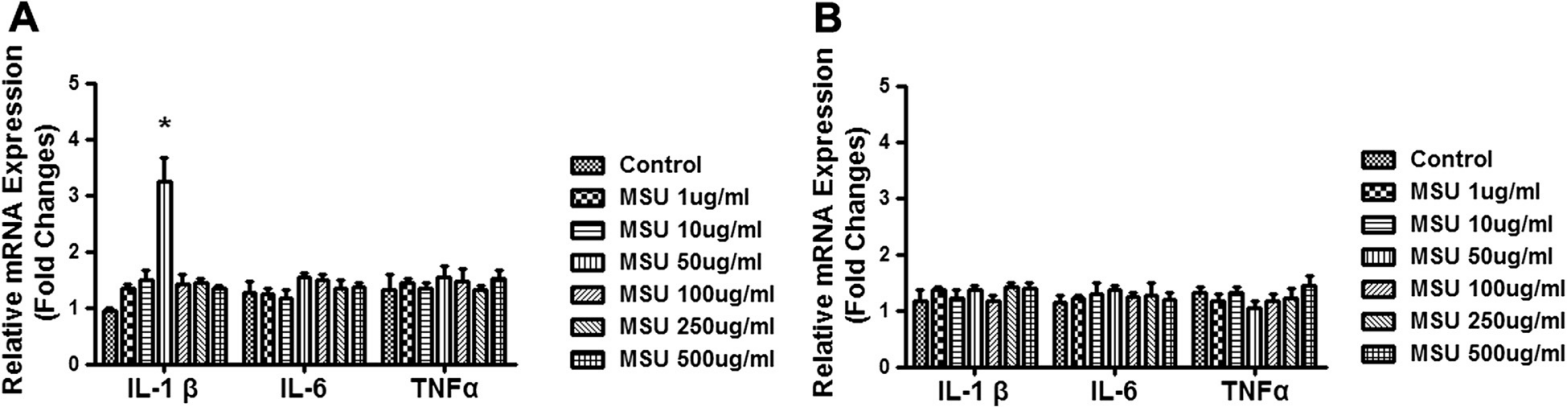
**MSU induced IL-1** **β** **mRNA expression in FLS.** FLS were stimulated with 1 ug/ml, 10 ug/ml, 50 ug/ml, 100 ug/ml, 250 ug/ml and 500 ug/ml of MSU at short-term time point 6 h and long-term time point 48 h respectively. Cells were collected for IL-1β, TNF-α and IL-6 mRNA detection by Q-PCR. The concentration of MSU 50 ug/ml induced the significant increase of IL-1β mRNA expression in the cells collected after 6 hours MSU exposure (**A**, p<0.05). There was no significant difference for the levels of IL-6 and TNF-α mRNA expression in the cells of FLS exposed to different concentration MSU whether at the 6 h time point or the 48 h time point. **(B)** Data were three independent experiments and presented are mean ± S.E.M. *represents P < 0.05 in comparison with control during statistical analysis.

The mRNA levels of IL-1β, TNF-α and IL-6 in FLS treated with different doses of MSU were measured with Q-PCR at two representative time points, short-term 6 h and long-term 48 h. As shown in Figure 2A, 50 ug/ml of MSU induced a 3 to 4-fold increase in IL-1β mRNA expression 6 h after treatment (Figure 2A, p<0.05), compared to the control group. There was no significant change in mRNA levels of TNF-α and IL-6 at this time point. At the 48 h time point, different doses of MSU did not alter mRNA levels of IL-1β, TNF-α or IL-6 (Figure 2B).

### MSU mediated the consumption of storage pro-IL-1β at the 6 h time point in the FLS

**Figure 3:**
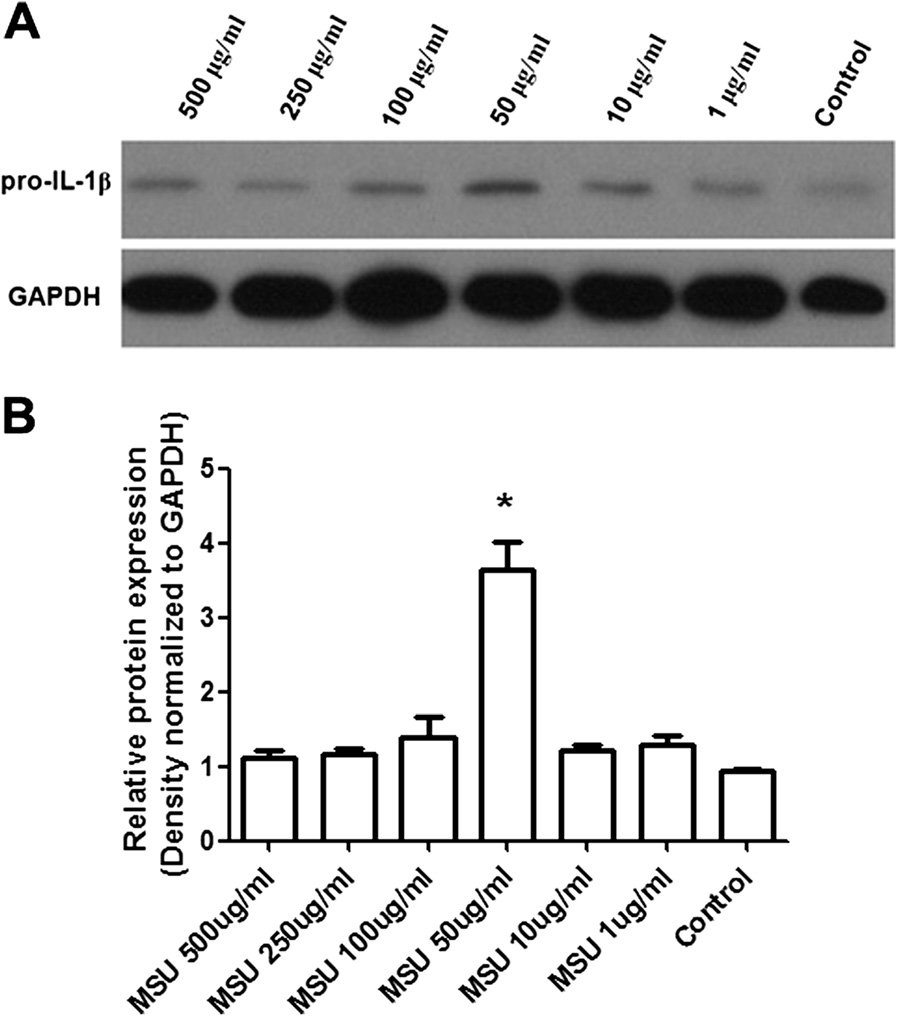
**Changes of pro–IL-1** **β protein level detected by Western blot in the cells of FLS.** Western blot analysis detected expected size protein band of pro–IL-1β **(A)**. The pro–IL-1β protein levels in different groups were expressed as a ratio to that of corresponding GAPDH **(B)**. Data presented are mean ± S.E.M. Data were three independent experiments. *represents P < 0.05 in comparison with control during statistical analysis.

Western blot analysis showed that MSU induced pro–IL-1β expression in FLS treated with different doses of MSU (Figure 3). A single band of the expected size (~31 kDa) for pro–IL-1β was detected by the specific primary antibody (Figure 3A). The increase in levels of pro–IL-1β protein was significant after 50 ug/ml of MSU treatment compared to the control group (Figure 3B; p<0.05). Also as shown in Figure 3B, the other doses of MSU had no effect on pro–IL-1β protein expression at the 6 h time point. At the 48 h time point, pro–IL-1β protein expression was undetectab. in all groups (data not shown).

### Effect of MSU on NLRP3 expression in FLS

**Figure 4:**
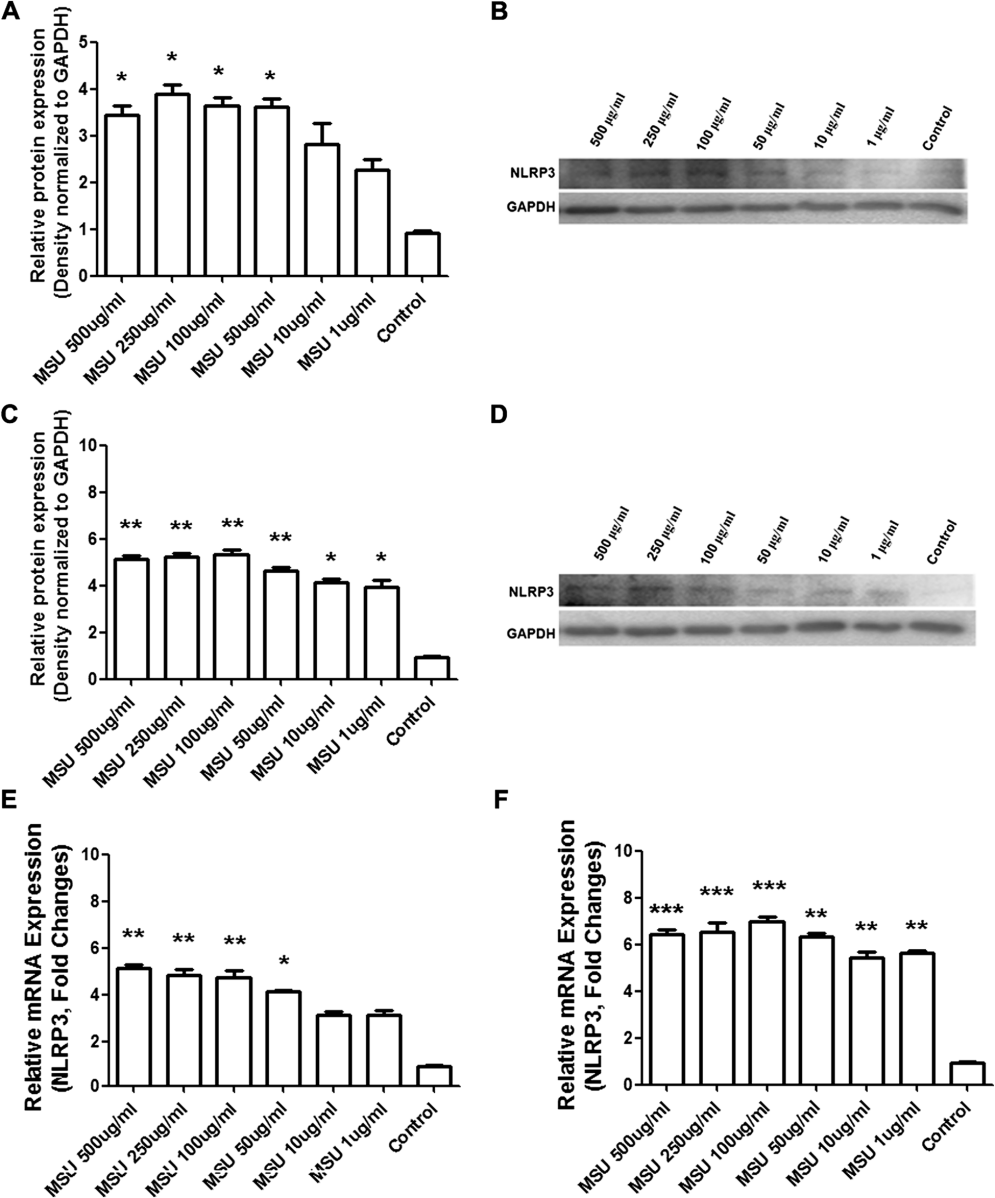
**Changes of NLRP3 protein level detected by Western blot and mRNA level detected by Q-PCR in the FLS.** FLS were stimulated with 1 ug/ml, 10 ug/ml, 50 ug/ml, 100 ug/ml, 250 ug/ml and 500 ug/ml of MSU at short-term time point 6 h and long-term time point 48 h respectively. Western blot analysis detected the NLPRP3 protein levels in different groups were expressed as a ratio to that of corresponding GAPDH at the 6 h time point **(A)**. The expected size protein band of NLPRP3 was shown at the 6 h time point **(B)**. At the 48 h time point, the NLPRP3 protein levels in different groups were expressed as a ratio to that of corresponding GAPDH **(C)**. The expected size protein band of NLPRP3 was shown at the 48 h time point **(D)**. There was significant difference for the levels of NLRP3 mRNA expression in the cells of FLS exposed to 50 ug/ml, 100 ug/ml, 250 ug/ml and 500 ug/ml concentration MSU at the 6 h time course **(E)**. The dramatically increase for the NLRP3 mRNA levels in the cells of FLS exposed to all concentration MSU at the 48 h time course were detected **(F)**. Data presented are mean ± S.E.M. Data are three independent experiments. *represents P < 0.05, **represents P < 0.01 and ***represents P < 0.001 in comparison with control during statistical analysis.

NLPR3 protein level was significantly increased following treatment with different doses of MSU at the 6 h time point (Figure 4A; p<0.05). The band of NLPR3 was detected by immunoblot (Figure 4B). In addition, no band was observed when the primary antibody was omitted (data not shown). Similar changes were observed at 48 h. At this time point NLPR3 protein level was dramatically increased after exposure to MSU (Figure 4C; p<0.05 and p<0.01) and the band of NLRP3 could still be detected (Figure 4D). According to the Q-PCR analysis, NLPR3 mRNA level was markedly increased in the FLS at 6 hours after exposure to different doses of MSU (Figure 4E; p<0.05 and p<0.01). Similarly, a significant increase in NLPR3 mRNA level was also seen at 48 h (Figure 4F; p<0.05, p<0.01 and p<0.001).

## Discussion

It is well-known that neutrophils and macrophages are involved in the inflammatory reaction in gout [19,22-24]. In addition, the role of inflammasomes in the development of a number of diseases including gout, has been demonstrated in these cells [17,23,25]. However, due to complex interactions among various cell types, including neutrophils, macrophages, mast cells, endothelial cells and synovial fibroblasts [26,27], it is possible that synovial fibroblasts also may play a role in modulating the inflammatory response to MSU crystals in gout. In this study, we addressed the role of synovial fibroblasts in mediating the release of mature IL-1β via activation of the NLRP3 inflammasome after exposure to MSU. We observed that MSU increased the release of IL-1β, but not IL-6 and TNF-a, in the supernatants of MSU-treated FLS. Low concentrations of MSU transiently triggered an increase in mRNA and protein levels of IL-1β in FLS. Moreover, the formation of the NLRP3 inflammasome in the FLS was also increased after short-term treatment (6 h) and long-term treatment (48 h) with MSU. Our findings collectively provide a new insight into the role of synovial fibroblasts in the pathophysiology of gout. They suggest that synovial fibroblasts may participate in the secretion of IL-1β via NLRP3 inflammasome formation, which could make synovial fibroblasts a potential therapeutic target for gout.

In rheumatoid arthritis (RA), synovial fibroblasts play an important role in the process of cartilage and bone erosion, presumably via the synthesis of inflammatory mediators, including chemokines [28,29], matrix metalloproteinases (MMPs) [30], and cytokines [31]. Peng et al. recently showed that MSU crystals upregulated cyclooxygenase 2 (COX-2) and interleukin 8 (IL-8) gene expression in human synovial fibroblasts [32]. Also in RA, elevated levels of miR-203 led to increased secretion of MMP-1 and IL-6 via the NF-κB pathway and thereby contributed to the activated phenotype of synovial fibroblasts [33]. In cultures of synovial cells from patients with RA, blocking TNF-α with antibodies significantly reduced the production of IL-1β, IL-6 and IL-8 [34]. Chen et al. showed that MSU, alone or in combination with TNF-α or IL-1β, were able to significantly increase the release of the IL-6, the chemokine CXCL8 and MMP-1 on the activation of human FLS from RA patients and normal control subjects [35]. Although this is an interesting study, they did not detect the release of IL-1β, by which was did in our present study. In summary, these studies suggest a potential role for synovial fibroblasts in MSU-induced inflammation. Our present study demonstrated that MSU induced a transient increase in the secretion of IL-1β, which indicates a potential role for FLS in the inflammatory pathophysiology of gout. However, no increase of IL-6 and TNF-a was detected in synovial fibroblasts after exposure to MSU. This may suggest that FLS play a different role in MSU triggered inflammation compared to macrophages and other types of immune cells. More studies are needed to further elucidate the mechanisms.

The concentration of MSU in our study ranged from 1 ug/ml to 500 ug/ml. It was lower than that used in other in vitro studies using macrophages or monocytes [36-38]. 50 ug/ml of MSU only induced mature IL-1 β secretion from FLS at the 6 h time point, which suggests that the effect of MSU on FLS is transient. Our results are supported by Margalit et al. [39], who showed that prostaglandins and other arachidonic acid metabolites, the inflammation mediation, are transiently formed after MSU crystal injection with peak levels occurring shortly after treatment. In the clinic, crystals are present, and may be retrieved by aspirating the synovial fluid (SF) of gout patients, during gout flares, but also during asymptomatic periods [40,41]. However, no correlation has been reported between the size, shape and numbers of crystals in the SF and the severity of inflammation [42]. A potential explanation for this may be that the relationship between crystal formation and inflammation is modified by individual genomic background. Further analysis of the factors that regulate the cellular response to inflammatory crystals may identify potential therapeutic targets for gout.

IL-1β is a well-known member of the IL-1 family and a highly inflammatory cytokine. The production and activity of IL-1β is tightly regulated in a multi-step process. IL-1β mRNA transcripts are rapidly expressed, and the precursor of IL-1β is synthesized based on the activation of Toll-like receptors (TLRs) or IL-1 signaling [43]. Our study shows that mRNA levels of IL-1β are consistent with pro-IL-1β protein expression in FLS exposed to MSU. In vitro studies in human monocytes demonstrate that the phagocytosis of MSU crystals induces the release of several cytokines, including IL-1 β, TNF-α, IL-8, and IL-6 [44,45]. Based on in vitro studies and in vivo animal models, gout has been identified as a prototypical IL-1 β-dependent disease, an observation that has also been confirmed by clinical studies, addressing the therapeutic effect of IL-1 blockage [8,46]. In addition, the current study suggests that the release of IL-1β by synovial fibroblasts also may be of importance in the pathophysiology of gout.

Pro-IL-1 β needs a second signal in order to maturate its active form, since pro- IL-1β is biologically inactive and its processing to the active, secreted form takes places inside the cytoplasm or in specialized secretory lysosomes [47]. NLRP3 and its adaptor protein, ASC, mediate caspase 1-dependent processing of certain cytokines, especially IL-1β [48,49]. There is clear evidence that MSU crystals trigger the activation of the NLRP3/ASC/caspase1 inflammasome, an effect that culminates in the production of IL-1β [17,50,51]. The present study showed increased mRNA and protein levels of NLRP3 in FLS exposed to MSU, which suggests that IL-1β secretion is regulated by the NLRP3 inflammasome in FLS.

It is well-known that the release of biologically active IL-1β needs two signals, which means the production of mature IL-1β is tightly regulated. On the one hand, transcription of the IL-1β gene and production of cytosolic pro–IL-1β are dependent on activation of NF-kB via, for example, TLRs. On the other hand, the second signal leads to cleavage of pro–IL-1β by NLRP3/ASC/caspase-1 and release of mature IL-1β [52-54]*.* The present study showed a transient increase in IL-1β and NLPR3 expression in FLS. Collectively, our data suggests that cleavage of pro- IL-1β to IL-1β is induced by the NLRP3 inflammasome. Migita et al. showed that MSU stimulation resulted in the activation of caspase-1 and production of active IL-1β and IL-1α in serum amyloid A (SAA)-primed synovial fibroblasts, and that the effect could be impaired in cells by silencing NLRP3 using siRNA or inhibition of caspase-1 [55]. Together with the results of the current study, these findings provide insight into the molecular processes underlying the synovial inflammatory condition of gout.

In addition, our present results showed that, even though the NLRP3 expression were upregulated throughout the whole range of concentrations and time points used, the most effects of MSU are only observed at 50ug/mL of 6 h time points, which is essentially in agreement with pro–IL-1β expression in the FLS cells. Recently, Choi and Ryter [56] summarized how the NLRP3 inflammasome is typically activated by the bimodal signaling pathway. A Toll-like receptor (TLR)-dependent priming step activates the NF-kB dependent transcription of NLRP3 and the pro-forms of the pro-inflammatory cytokines (i.e., IL-1β). The activation of the P2X7R receptor by stimulation with exogenous ATP, which triggers potassium ion (K+) efflux, is the second signal. Besides that, the NLRP3 inflammsome may be activated by agents that cause mitochondrial dysfunction and the particulates such as monosodium urate or silica. Based on these possible reasons, although the most effects of MSU are only observed at 50ug/mL of 6 h time points, the NLRP3 expression in our present study may be existed throughout the whole range of concentrations and time points we used. It is currently unclear how the results of relatively short term expression in the FLS relate to IL-1βand pro-IL-1β levels in a condition characterised by continuing MSU exposure during gout.

## Conclusions

To our knowledge, the current study is the first to demonstrate the essential role of the NLRP3 inflammasome in synovial fibroblasts in the pathogenesis of gout. MSU activated the NLRP3 inflammasome in FLS, which led to the processing and maturation of pro–IL-1β into the active form of IL-1β. MSU-induced production of IL-Iβ was transient and partially dependent on NLRP3 inflammasome activation. It is hypothesized that a transient increase in IL-1β production may enhance inflammation and then transfer the inflammatory reaction to other cells. The results indicate that targeting the NLRP3 inflammasome in FLS may be a relevant therapeutic strategy in the treatment of gout.

## Additional file

Additional file 1: Figure S1. MSU induced IL-1β production in FLS. FLS were stimulated with 10 ug/ml, 25 ug/ml, 50 ug/ml, 75 ug/ml and 100 ug/ml of MSU at 6 h and 48 h respectively. The control group is treated with PBS, which is used to dilute different dosage of MSU. Supernatants were detected for IL-1β protein by ELISA. The concentration of MSU 50 ug/ml induced the significant increase of IL-1β in the supernatants collected after 6 hours MSU exposure (A, p<0.05). There is no significant difference at the 48 h time point (B). Data presented are mean ± S.E.M. *represents P<0.05 in comparison with control during statistical analysis.

## Abbreviations

MSU: Monosodium urate
IL-1β: Interleukin-1β
FLS: Fibroblast-like synoviocytes
NLRP3: Nucleotide-binding domain-like receptor protein 3
ASC: Apoptosis-associated speck like protein
MCP-1: Monocyte chemoattractant protein-1
RA: Rheumatoid arthritis
MMPs: Matrix metalloproteinases
COX-2: Cyclooxygenase 2
TLRs: Toll-like receptors
SAA: Serum amyloid A
