# Supplementary figures and images for: Role of the NLRP3 inflammasome in the transient release of IL-1β induced by monosodium urate crystals in human fibroblast-like synoviocytes
Source: J Inflamm (Lond). 2015 Apr 10;12:30. doi: 10.1186/s12950-015-0070-7 (PMC4403983; doi:10.1186/s12950-015-0070-7)

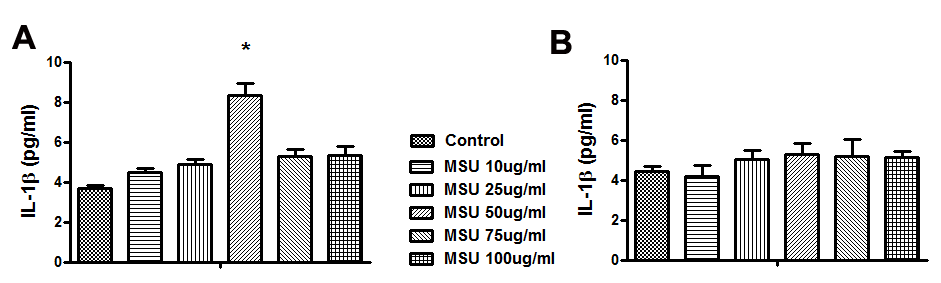

Supplement: Additional file 1: Figure S1. — MSU induced IL-1β production in FLS. FLS were stimulated with 10 ug/ml, 25 ug/ml, 50 ug/ml, 75 ug/ml and 100 ug/ml of MSU at 6 h and 48 h respectively. The control group is treated with PBS, which is used to dilute different dosage of MSU. Supernatants were detected for IL-1β protein by ELISA. The concentration of MSU 50 ug/ml induced the significant increase of IL-1β in the supernatants collected after 6 hours MSU exposure (A, p<0.05). There is no significant difference at the 48 h time point (B). Data presented are mean ± S.E.M. *represents P<0.05 in comparison with control during statistical analysis. [file 12950_2015_70_MOESM1_ESM.tiff]
